# Esophageal reconstruction using a hypopharyngeal anastomosis – a single center experience with review of the literature

**DOI:** 10.1186/s13019-025-03575-8

**Published:** 2025-08-26

**Authors:** Lauren Bougioukas, Lucio M. Pereira, Matthew J. Weiss, Lawrence R. Glassman, David Zeltsman, Kevin M. Hyman, Julissa E. Jurado, Paul C. Lee

**Affiliations:** Northwell, New Hyde Park, NY USA

**Keywords:** Hypopharyngeal anastomosis, Esophageal reconstruction, Lye, Corrosive ingestion, Pharyngeal anastomosis

## Abstract

**Background:**

Lye ingestion or other esophageal trauma may require surgical reconstruction. The hypopharyngeal anastomosis during esophageal reconstruction is a technically demanding procedure with many nuances in approach. Patients often have a challenging post-operative course, and few regain the ability to tolerate a normal diet.

**Case presentation:**

We describe a case series of three patients (2 colon interpositions and 1 gastric pull-up) who underwent esophageal reconstruction with a hypopharyngeal anastomosis at our institution from years 2017 to 2024, then review the literature.

**Conclusions:**

We recommend a multidisciplinary team approach with otolaryngology and/or general surgery for the neck dissection and preparation of the conduit. For the hypopharyngeal anastomosis, we recommend a two-layer, interrupted suture method to the left, lateral piriform sinus along with careful laryngeal nerve preservation. Patients require close follow-up for endoscopic therapy to treat the often-inevitable dysphagia after surgery. All three patients survived and had improved swallow function after surgery.

**Supplementary Information:**

The online version contains supplementary material available at 10.1186/s13019-025-03575-8.

## Background

Extensive esophageal injury in the form of lye ingestion or trauma may require surgical esophageal reconstruction. There are various approaches to reconstruction when conservative treatment fails: the new conduit can come from the stomach, jejunum, or colon; the anastomosis can be stapled or sutured; and the location of the anastomosis can involve the pharynx or colon. The most common approach is an esophageal-colonic anastomosis, where an anastomosis is created between the cervical esophagus and the new conduit [1]. However, in a small fraction of patients, when there is an inadequate amount of viable cervical esophagus, a pharyngeal anastomosis is needed [2]. The hypopharyngeal anastomosis has been previously described using a one- or two- interrupted suture method [1–3, 15–17]. Herein, we describe three patients (2 colon interpositions and 1 gastric pull-up) who successfully underwent a hypopharyngeal anastomosis at our institution from years 2017 to 2024. Preoperatively, one patient’s larynx demonstrated post-traumatic changes from caustic ingestion with scar tissue and fibrosis; otherwise, the other larynxes were not injured. In our approach, a two-layered interrupted suture method was used to the left, lateral piriform sinus with careful nerve protection. All 3 (100%) patients survived and had improved swallow function; 2 (66.7%) patients required post-operative balloon dilation; 1 (33.3%) patient required revision of the anastomosis; and 2 (66.7%) patients developed dysphonia.

## Case presentation

### Case #1

A 33-year-old male, who is a poor historian had an accidental ingestion of grill cleaner seven years prior to presentation. The patient had multiple surgeries, including a substernal colonic interposition graft sewn with a cervical esophago-colonic anastomosis and Roux-en-Y colonic jejunal anastomosis with J-tube placement at an outside hospital. On presentation to our clinic, the patient was receiving tube feeds for 14-hours per day and had fusion of the posterior pharyngeal wall obliterating the entrance to the esophagus on direct laryngoscopy. The larynx appeared normal.

**Fig. 1:**
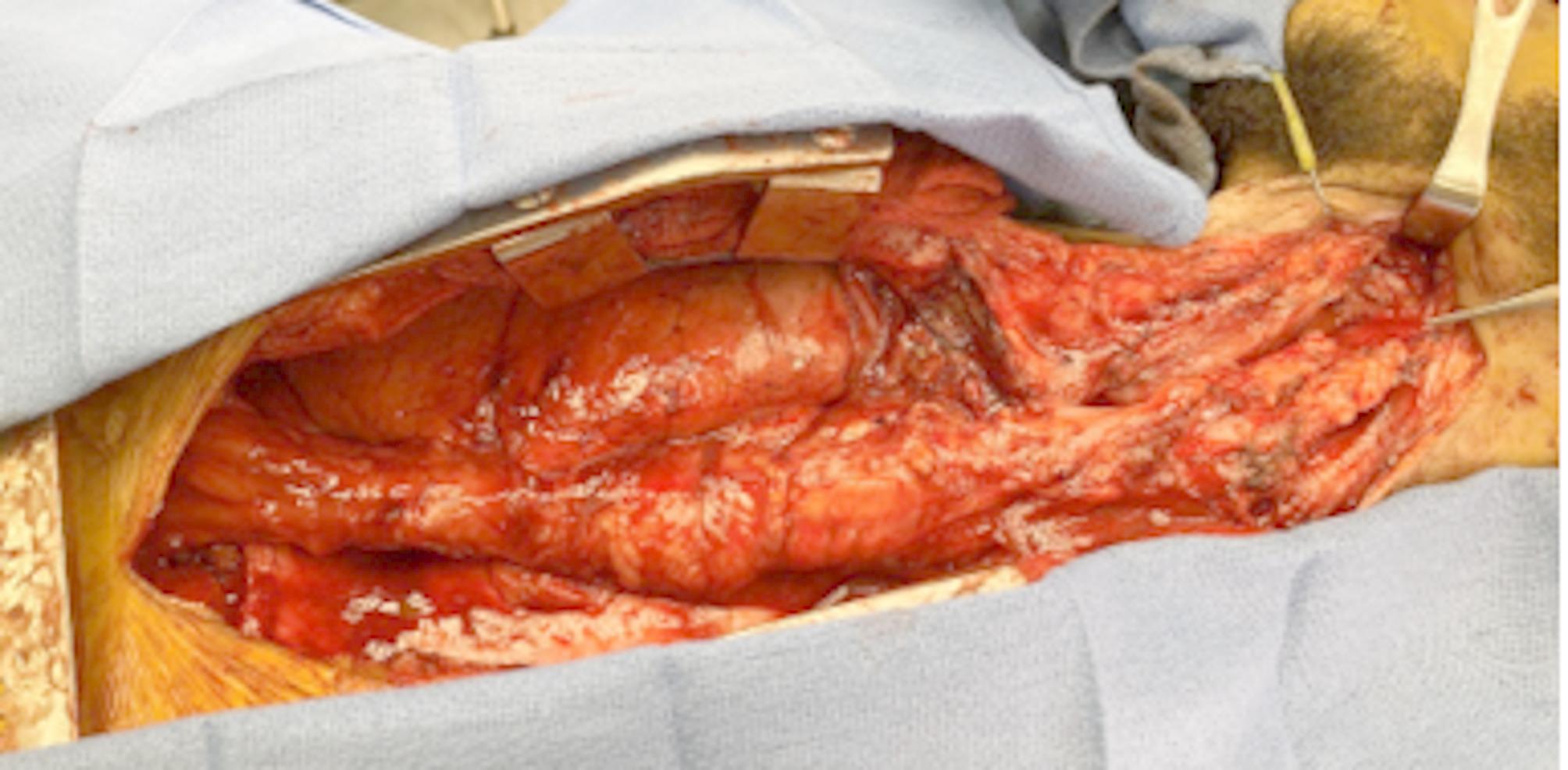
Intra-operative image after median sternotomy, showing the previous colon conduit

The patient was taken to the operating room for revisional surgery. The esophageal conduit already in place appeared viable. A median sternotomy with blunt dissection was used to mobilize the colon for the hypopharyngeal anastomosis (Fig. 1).

During the procedure, the recurrent laryngeal nerve was preserved. The superior laryngeal nerve was sacrificed since it was in the perfect area for the anastomosis. A Deaver retractor was introduced into the oral cavity and placed inside the piriform sinus on the left side to improve exposure. The adventitia of the colon was stitched to the inferior constrictor posteriorly and then the mucosa was reconstructed with 2 − 0 Vicryl horizontal mattress stitches invaginating the mucosa into the lumen. Once the posterior wall was reconstructed, a nasogastric tube was placed through the anastomosis and into the inferior portion of the thoracic colon. The anterior wall was then reconstructed in the same fashion with 3 − 0 Vicryl horizontal mattress stitches with invagination of the mucosa. A second layer was then performed anteriorly between the adventitia and the inferior constrictor muscle. Another layer was placed posteriorly to reinforce the anastomosis and prevent any tension in the mucosa.

One-month after surgery, a modified barium swallow demonstrated complete obstruction at the level of the piriform sinus with no passage into the neo-esophagus, and upper endoscopy showed no communication between the pharynx and colon. The patient was taken back to the operating room for revision of the anastomosis. After mobilization of the colon, the area of attachment to the piriform sinus was identified. The submandibular gland made access to this area difficult, so the submandibular gland was dissected off the facial artery and veins. The lingual and hypoglossal nerves were identified and preserved. The colon was dissected off the piriform sinus and there was virtually no opening at the level of the piriform sinus. The colon was inspected, and the mucosa looked viable and healthy. A Deaver retractor was placed into the patient’s oral cavity with the tip protruding over the piriform sinus. The inferior constrictor muscle was completely dissected off the mucosa in that area and the mucosa was then opened, and a 2 cm opening was made in the left piriform sinus. The posterior wall of the anastomosis was then made using 3 − 0 Vicryl horizontal mattress stitches.

Once the posterior wall was reconstructed, a salivary bypass tube was placed into the oral cavity and introduced into the colon. Once it was in place in the piriform sinus and going through the anastomosis, the anterior wall of the reconstruction was reconstructed again using 3 − 0 Vicryl horizontal mattress stitches. The adventitia of the colon was then stitched to the surrounding tissues around the anastomosis, mainly the inferior constrictor muscle as a second layer, and the wound was irrigated, and the procedure was completed.

Upper endoscopy was performed a month later showing a patent anastomosis. Five-months after revisional surgery, the patient was swallowing normally, gaining weight, and tolerating a diet. His feeding tube was removed, and he did not have any post-operative laryngeal or voice complaints.

### Case #2

A 60‑year‑old male, who was a former smoker, with a past medical history of hypertension and diabetes, underwent a cervical spinal fusion seven years prior to presentation. His post-operative course was complicated by erosion of the anterior cervical spinal plate through the esophagus. The patient underwent neck exploration, removal of infected hardware, debridement of the neck as well as resection of a Zenker’s diverticulum. Nine months prior to presentation, the patient developed a chronic hypopharyngeal cutaneous fistula and an esophageal stricture.

The patient was taken to the operating room. The right chest was prepped. The robotic Xi system was used to dissect and mobilize the esophagus from the diaphragm and airway. There was a large inflammatory phlegmon in the esophagus. The procedure was converted to open with a posterolateral thoracotomy incision. The esophagus was further mobilized from the airway and spine using sharp and blunt dissection. Next, the hypopharyngeal cutaneous fistula and cervical esophagus were mobilized. A laparotomy incision was made. A pyloromyotomy was performed and the stomach was made into a 2.5‑3 cm gastric tube based on the greater curvature of the stomach. The previous PEG tube was also excised. Next, a feeding J-tube was placed, and the tip of the gastric conduit was pulled to the neck. For the hypopharyngeal-gastric anastomosis, the posterior wall of the stomach was stitched to the posterior mucosa of the pharynx with 3 − 0 Vicryl horizontal mattress stitches with the knot inside to invert the edges of the mucosa. Another row of horizontal mattress stitches was placed in the anterior wall with complete reconstruction and anastomosis of the stomach to the pharyngeal mucosa. A second layer of 3 − 0 Vicryl stitches was placed in a running fashion for the anterior wall, and in an interrupted fashion for the posterior wall to help invaginate the tissues even more. During the reconstruction, a nasogastric tube was placed and stitched to the columella of the nose.

Postoperatively, the patient had some dysphonia. Laryngoscopy demonstrated vocal cord paralysis that resolved in a few weeks. He can swallow but has had some ongoing reflux and delayed gastric emptying, which is being treated with dilation of the pylorus, Botox injections, and medical management. His feeding tube has been removed.

### Case #3

A 30-year-old male with past medical history of social cigarette smoking and use of vaping products and marijuana, had an accidental lye ingestion at age two. The patient underwent a right colonic interposition at age four. The patient had complete dehiscence and breakdown of that colonic interposition (both the colonic-hypopharyngeal and colonic-gastric anastomoses). The previous procedure was also complicated by tracheal strictures (with tracheostomy, now decannulated). The patient had been unable to tolerate any food for greater than twenty years and had been gastrostomy-tube dependent for over twenty-eight years. He came to the clinic with hopes of being able to eat.

Preoperatively, the patient underwent upper endoscopy and laryngoscopy which showed no opening into the esophagus or pharynx; however, the pylorus was patent. The larynx exhibited post-traumatic changes from caustic ingestion with scar tissue and fibrosis. The patient had a colonoscopy which showed possibility of reconstruction using the left colon. A team of surgeons from thoracic surgery, otolaryngology, and general surgery planned to perform the procedure. Plastic surgery was on standby should a supercharged jejunal conduit be needed.

**Fig. 2:**
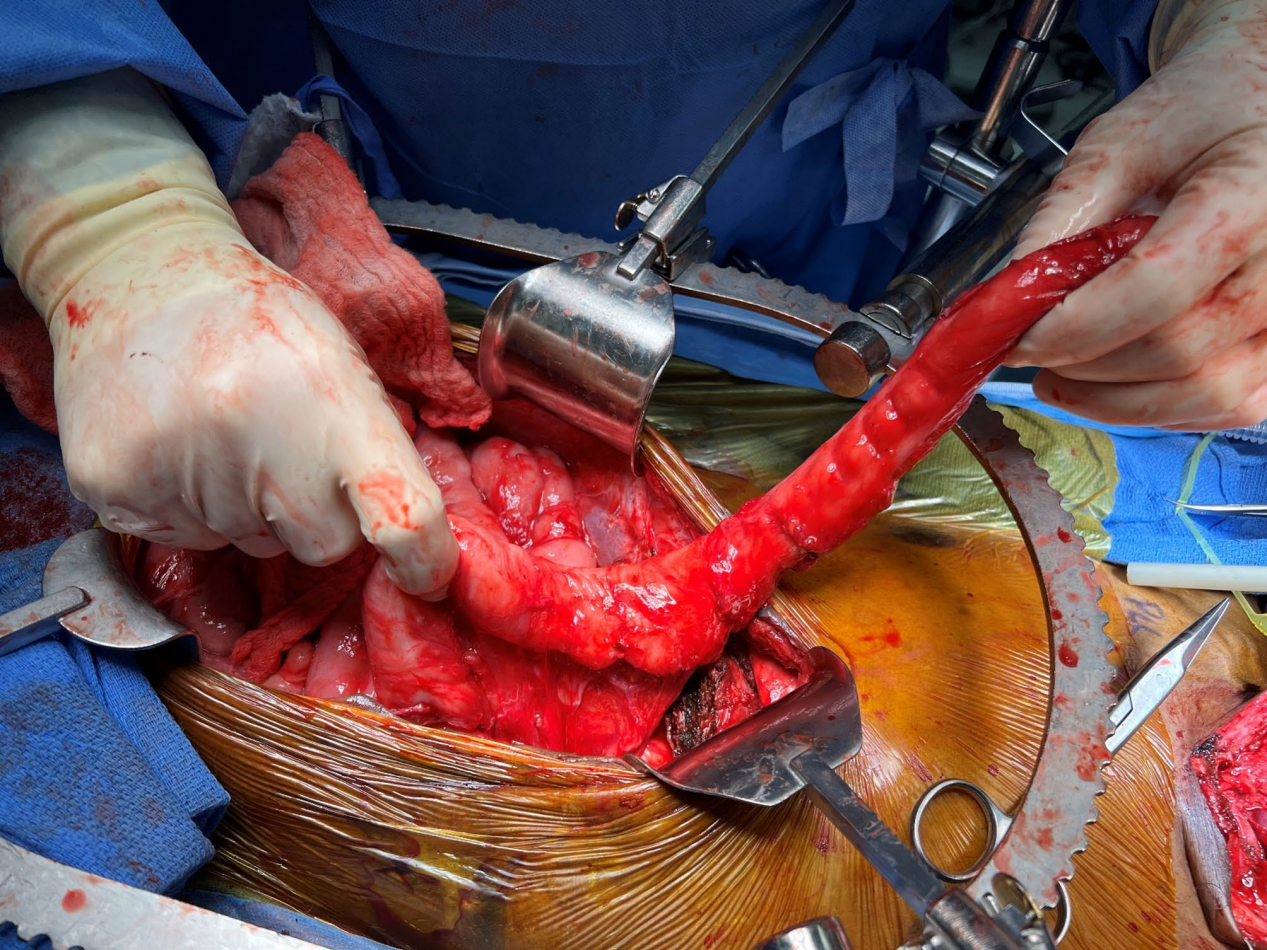
Left colon harvest

The patient was taken to the operating room, where he received an epidural. A laparotomy was performed, and dense adhesions were lysed. The previous gastrostomy tube site was closed. The left colon was mobilized to the ileum, which was divided at the ileocolonic anastomosis (Fig. 2).

All vessels that were to be divided were tested with bulldogs, and pulses were visible and bounding. The high rectal area or low sigmoid colon was transected.

At the neck, a hockey stick incision was made on the left side with vertical extension into the manubrium to access the sternoclavicular joint. The piriform sinus was mobilized. Part of the manubrium on the left side and head of the left clavicle were resected to allow mobilization of the colonic graft. The colon was dissected and brought through the substernal space into the neck. The colonic graft was short by 2–3 cm, mostly due to an abnormally enlarged liver hindering the adequate mobilization of the colonic graft. The conduit was taken back to the abdomen, where the left lateral portion of the liver was removed to create more room for the colonic conduit. The left colon was brought through the gastrohepatic ligament on its pedicle to achieve an additional 2–3 cm of length on the colon.

**Fig. 3:**
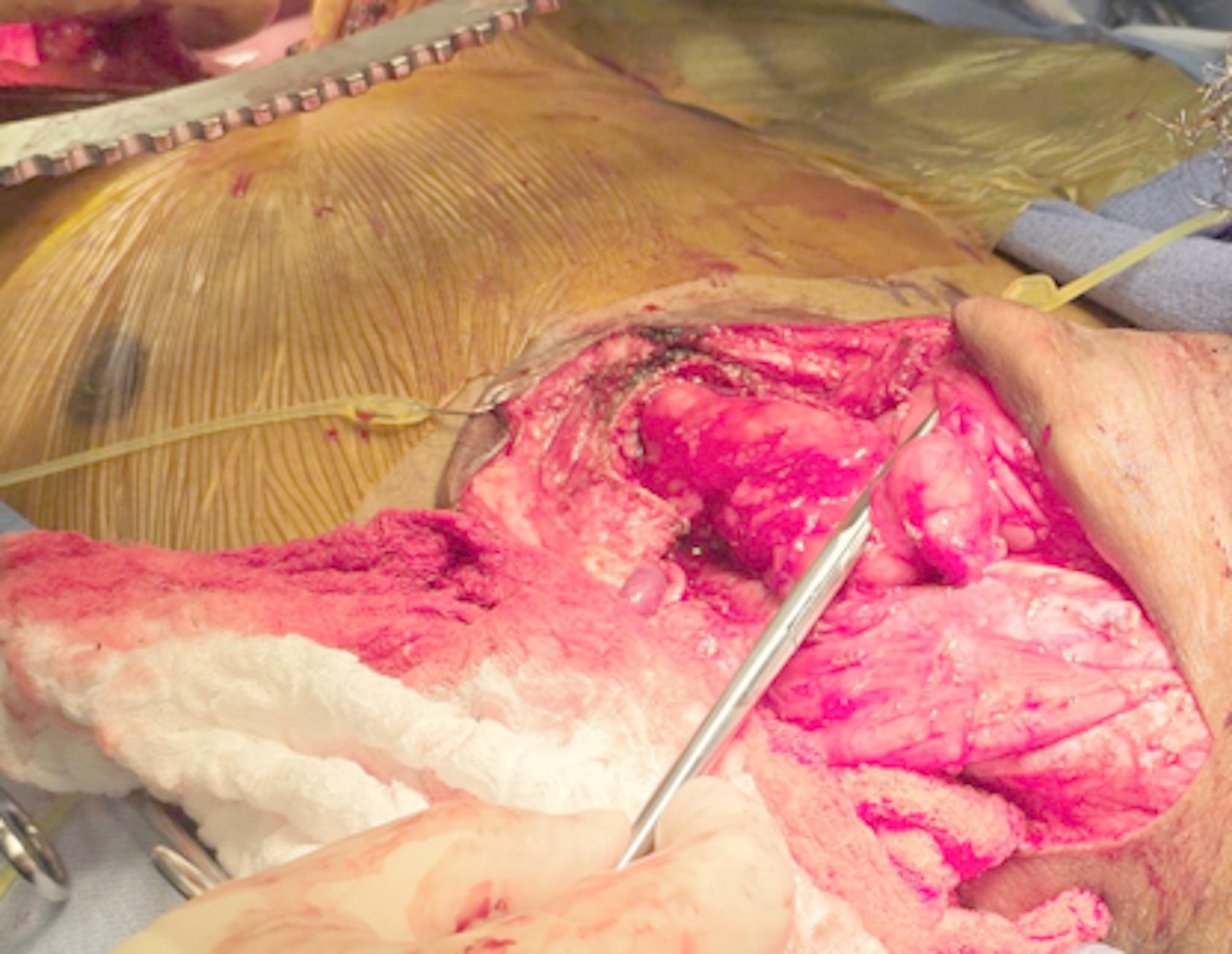
Metzenbaum scissors showing the left, lateral border of the piriform sinus, the location of the hypopharyngeal-colonic anastomosis

The piriform sinus was exposed by removing some of the fibers of the inferior constrictor muscle on the left side, being careful to preserve the superior laryngeal nerve (Fig. 3).

**Fig. 4:**
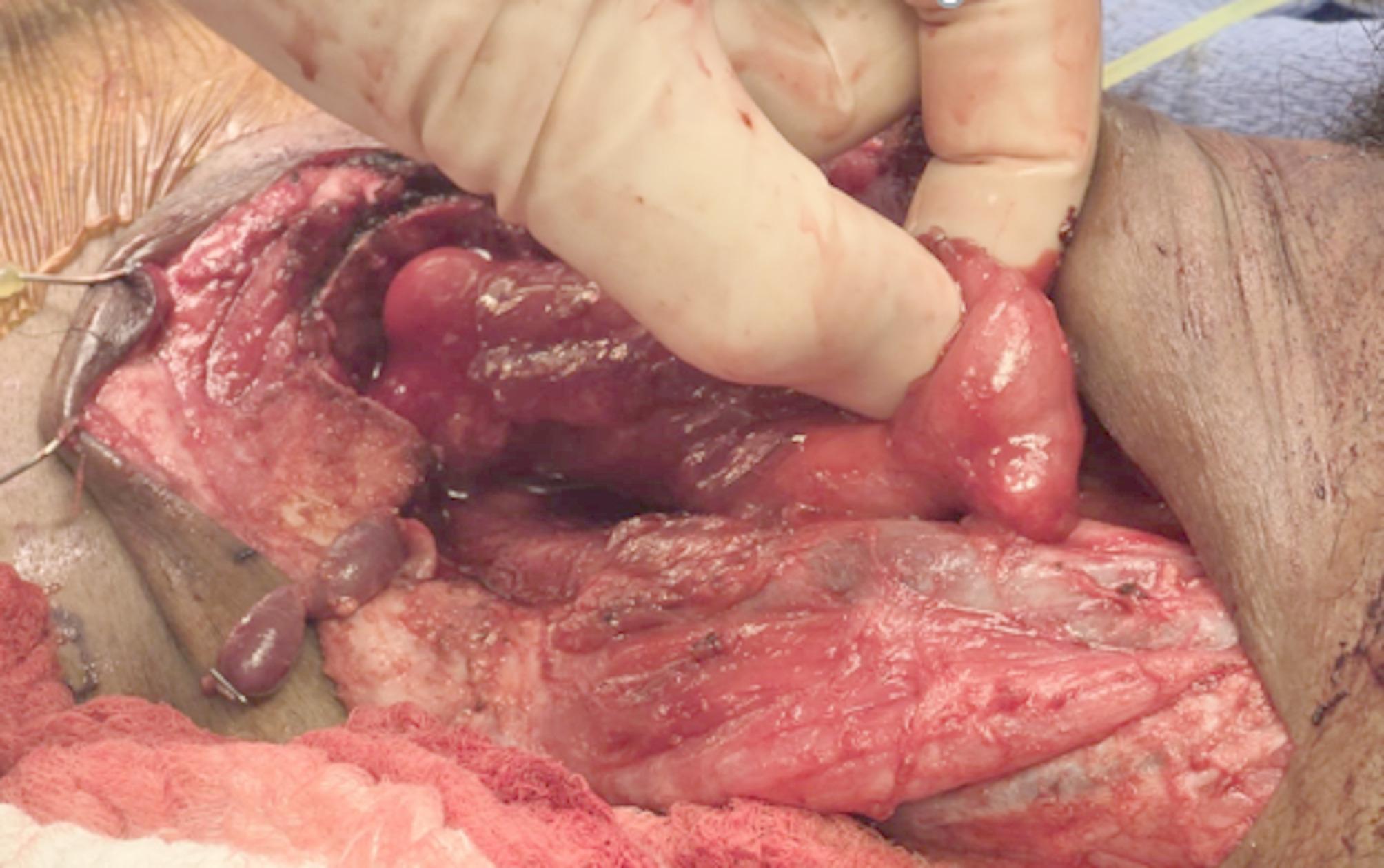
Completed hypopharyngeal-colonic anastomosis, with left colon anastomosed to the left lateral wall of the piriform sinus

A Deaver retractor was introduced through the mouth and placed in the left piriform sinus to improve the visualization of the mucosa in that area. A 2 cm incision was made in the mucosa of the piriform sinus and another 2 cm incision was made in the colon. The posterior wall of the anastomosis was then performed with interrupted 3 − 0 Vicryl. The mucosa was then approximated with 3 − 0 Vicryl. The anterior wall was then approximated with 3 − 0 Vicryl for the mucosal layer and 3 − 0 Vicryl for the 2nd layer, approximating the serosa of the colon to the inferior constrictor muscle (Fig. 4).

Additionally, the cologastric anastomosis was done in two layers with running 3 − 0 PDS suture, and the ileocolonic anastomosis to the high rectum-low sigmoid colon was completed with running 3 − 0 PDS suture in two layers as well. At the end of the procedure, a J-tube was placed.

Postoperatively, the patient has improved swallow function. He has required balloon dilation of the hypopharyngeal anastomosis x 6 and supplemental tube feeds to support nutritional goals. In addition, he has some postoperative dysphonia. Fiberoptic laryngoscopy demonstrated the presence of scar tissue on the supraglottic larynx without signs of obstruction. The patient is currently undergoing speech and swallow evaluation and rehabilitation.

## Discussion and conclusions

Esophageal reconstruction involving the hypopharynx is complicated, as the hypopharynx is involved in the process of respiration and nourishment [4–7]. The hypopharyngeal anastomosis can be made especially challenging in settings of malnutrition and radiation [7–10]. Most esophageal reconstructions are performed with an esophageal-colonic anastomosis. There is a paucity of studies describing the hypopharyngeal anastomosis; a general overview of these studies is shown in Table 1. When the anastomosis is successful, patients recover the ability to eat, speak, and breath; however, often the anastomosis is associated with complications requiring long-term treatment [5–7, 11].

**Table 1:** General overview of hypopharyngeal anastomosis studies, table design derived from Sanchez, et al (2022). NR (Not recorded).

Author	Period	# Patients with Hypopharyngeal Surgery	Method of Injury	Endoscopic Dilation	Time to Surgery from Injury	Conduit (iso-peristaltic, anti-peristaltic)	Conduit perfusion	Route	Type of Anastomosis	Mortality	Tolerating normal diet?	Regain Swallow Function?	Still using feeding tube?
Chilgar, et al	Jan 1994 to December 2012	94	Hypopharyngeal Cancer, Squamous cancer	NR	NR	Free colon or ileo-colon iso	NR	NR	2-layered suture vs. modified 2-layer suture with distal conical trimming	NR	NR	NR	NR
Jiang, et al	Aug 1988 to June 2023	14	Caustic injuries	NR	17.5 months	Left colon, anti	Mesenteric angiography, temporary occlusion	Mediastinum	1-layered suture technique (prox) 2-layered suture technique (distal)	NR	14/14	NR	NR
Wu, et al	Sept 1976 to April 2000	50	Caustic injuries	NR	37 (74%) operated on within 6 months	28/50 with ileocolon	Temporary clamping	44/50 substernal	1 or 2-layered interrupted sutures above level of abnormal mucosa	1/50	NR	42/50	NR
Lu, et al	March 2003 to May 2006	5	Stage IVA hypopharynx cancer	NR	NR	Left colon with distal arterial enhancement (superior thyroid artery in 4; facial artery in 1)	Visual	Posterior mediastinum	NR	0	All	All	NR
Tannuri and Tannuri	Feb 1982 to Dec 2015	19	Caustic	Yes	NR	Transverse and right colon; isoperistaltic	NR	Retrosternal, unless esophagectomy done where posterior mediastinum was used	1-layer interrupted	0	All	All	All
Denewer, et al	Jan 2004 to Dec 2012	142	Most squamous cell	NR	NR	Including 3 revisional surgeries; pectoralis flap in 48, jejunal flap in 28, augmented colon bypass in 4, gastric pull-up in 32, gastric tube in 30	NR	NR	NR	15/142	Including 3 revisional surgeries: 40% of the pectoralis flap; 80% jejunal flap; 75% of augmented colon bypass; 82.4% with gastric pull-up; 86.7% with gastric tube	NR	NR
Bisquera, et al	~ 2022	1 (oropharynx)	Caustic ingestion	No- complete obliteration	~ 6 months	Left colon	Temporary clamping	Subcutaneous	Two-layered suture technique proximal; Roux-en-Y	No	Yes	Yes	No
Pegan, et al	10-year period	31	Hypopharyngeal carcinoma	0	NR	Radial forearm flap in 9; jejunum in 7, gastric tube in 15	NR	NR	NR	0%	29/31	29/31	2/31
Tran and Celerier	1978–1986	18	Caustic	NR	4 months- 28 years	Right ileocolon in 17; transverse colon in 1	NR	Retrosternal	NR	0 (3 died from severe malnutrition)	11	11	4/14
Marzouki, et al	~ 2021	1	Hypopharyngeal cancer, squamous cell	NR	NR	Tubed radial forearm flap	NR	NR	NR	No	Yes	Yes	No
Schultz, et al	~ 2018	8	NR	NR	NR	Forearm flap and tubed pectoralis major flap	NR	NR	Prox: Vicryl suture; Distal: Vicryl suture and 21mm diameter circular stapler with 2 rows of staples	0	NR	5/5 forearm	NR
Wang, et al	May 1995 to Nov 2021	75	Hypopharyngeal cancer	NR	NR	Stomach	NR	Posterior Mediastinum	Manual in 47; mechanical in 28 via circular stapler	4	NR	NR	NR
Zeng, et al	Jan 2005 to March 2017	20	Corrosive	NR	6 months to 45 years	Colon	Palpation temporary occlusion	Retrosternal	End-to-end manner with single-layer and broad-border hand sewn with delayed absorbable suture; distal end with continuous suture	0	NR	NR	NR
Choi, et al	~ 1997	7	Alkali and acid	Yes	6 months-10 years	Colon, radial forearm flap, right shoulder pedicle flap	NR	NR	NR	0	3	5	4
Zangi, et al	2009 to 2014	9	Corrosive	NR	4–10 months	Left colon isoperistaltic	Temporary clamping	Retrosternal or Posterior Mediastinum	Single layer of interrupted Vicryl	3	NR	6	NR
Pesko, et al	January 1978 to January 2004	40	Hypopharyngeal carcinoma	NR	NR	Stomach in 29; colon in 11	NR	NR	NR	6 (13%)	NR	NR	NR
Inoue, et al	~ 1992	28	Hypopharyngeal carcinoma	NR	NR	Jejunum with microvascular anastomosis	Angiography preop	NR	NR	NR	NR	NR	NR

**Table 2:**
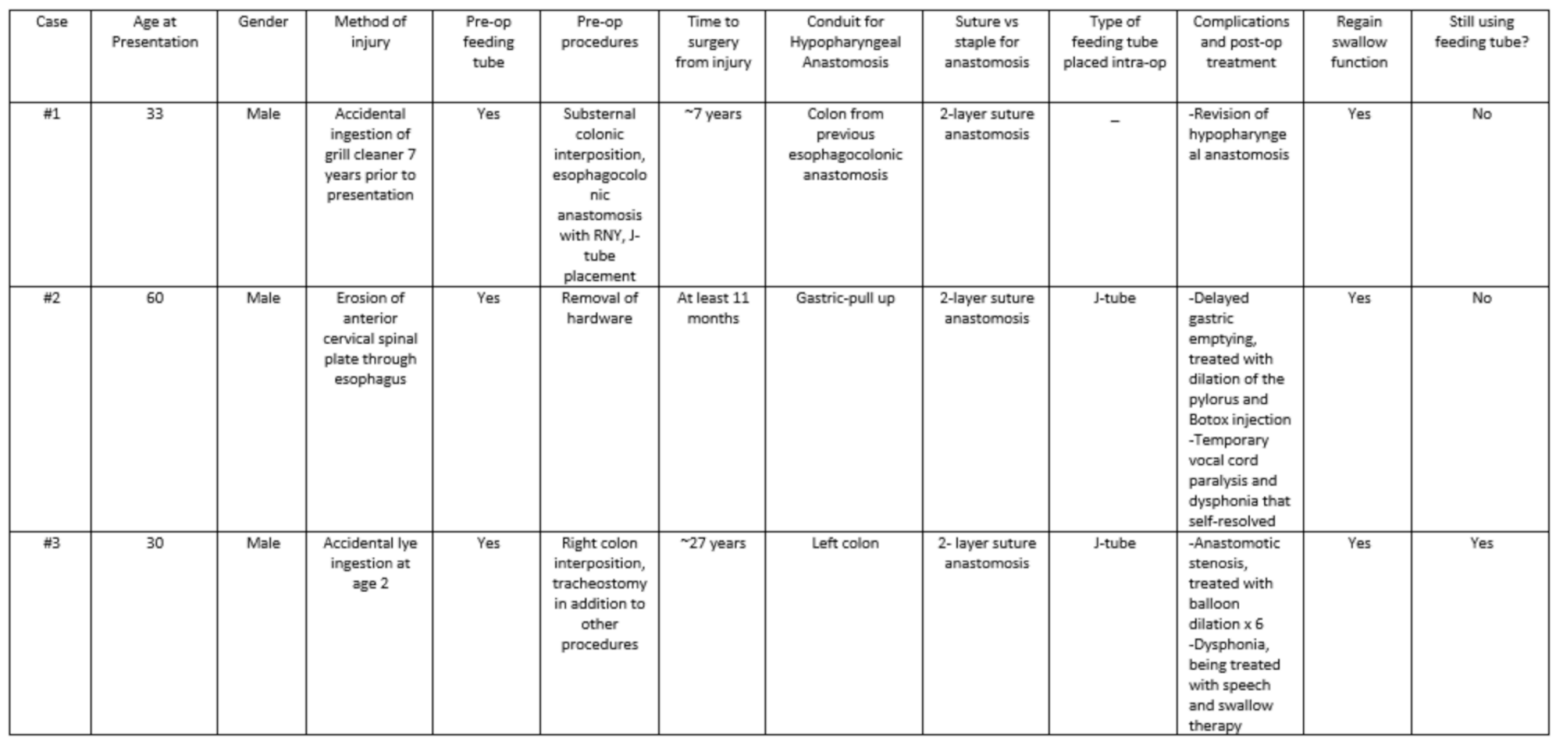
Summarizes the demographic and clinical details of the three patients, who had a hypopharyngeal anastomosis during esophageal reconstruction

The hypopharynx can become injured with alkaline and acidic substances resulting in liquefactive necrosis or coagulative necrosis, respectively [1, 2, 5, 11–16]. Oftentimes these causes are suicidal or accidental [5, 12, 15]; and these ingestions more commonly occur in males as witnessed in this case series [1, 2, 4, 16, 17]. Other etiologies that may cause injury to the hypopharynx include hot liquid and hypopharyngeal carcinoma (most common type is squamous cell carcinoma) [2, 4, 6, 9, 10, 18].

Endoscopic management with dilation is the first-line treatment [11–15]. However, these strictures tend to be associated with a high failure rate [16]. Scar formation starts in the first few weeks after injury and usually lasts for about six months, sometimes two years [12, 16]. The tendency to form stenoses decreases with time but may never cease, which can cause several trips to the endoscopy suite as seen in the cases above [5]. The chance of a successful surgery is thought to be greatest if the operation does not occur in the first six months to avoid stricture formation and surgery on these active areas [12, 14]. However, surgery may be warranted prior to six months if there is perforation, bleeding, or infection [11–15].

Prior to surgery, the patient undergoes rigorous work up and evaluation. The pharynx and esophagus are examined directly via upper endoscopy and direct laryngoscopy. In addition to endoscopic dilation, steroids can be given to decrease the formation of stricture; however, they may have an adverse impact on wound healing [12]. Swallow function can be assessed using a barium contrast study and the colonic conduit can be assessed via colonoscopy and angiogram [2, 14–16, 19]. In addition, conduit perfusion is assessed intraoperatively via visualization, palpation, and selective clamping [20]. In cases of suicide, psychiatry must evaluate the patient for stabilization [5, 15, 16]. Tracheostomy may be needed in cases of respiratory distress [16]. A feeding tube is typically placed to optimize nutrition and a nasogastric tube can be placed to decompress the stomach [1, 2, 15]. In the days leading to surgery, the patient is given a clear liquid diet and bowel prep to prepare the conduit [2, 14, 17].

As seen in this case series, there are various options for conduit, including the stomach, colon, jejunum, muscle, and skin flaps [4, 6, 15] (Table 2). This portion of the procedure may be augmented with help from general surgery. The gastric pull-up is commonly used, seen in Case 2. It only has one anastomosis and is less technically demanding compared to the other options; however, often it is not a suitable conduit due to inadequate length, risk of long-term gastroesophageal reflux disease, and injury in the case of caustic ingestion [2, 11, 12, 18, 21]. The colonic conduit (right colon, transverse colon, left colon) is preferred for reconstruction after caustic ingestion due to its long length, acid resistance, and vascular supply; however, it is associated with a higher chance of leak due to having two anastomoses [1, 7, 12]. The right colon has the ileocecal valve which can help prevent regurgitation and has a better size match at the proximal anastomosis; however, a worse size mismatch at the distal anastomosis [6, 15]. Jejunal flaps are more commonly associated with fistulas at the proximal anastomosis due to size mismatch of the conduit compared to the pharynx and its finer vasculature [12, 19]. If the jejunal conduit is utilized, a “supercharge procedure” or “arterial enhancement” with microvascular vascular anastomoses can be performed [9, 18, 19]. Other conduits, though less commonly used, include the pectoralis major flap and radial forearm free flap [4, 9].

The conduit can be transposed through different sites to reach the hypopharynx, which include the posterior mediastinum, subcutaneous, retrosternal, transthoracic, and trans-pleural spaces [13, 14]. The posterior mediastinum is the shortest and can be utilized if the native esophagus is removed; however, if there is severe fibrosis and adhesions, the native esophagus can be left in place [1, 2, 15]. The next best option is retrosternal or substernal tunneling. However, if a tracheostomy is planned, the subcutaneous route must be utilized which requires the longest length of conduit but is associated with minimal dissection and shorter operative times [12, 14, 15]. The otolaryngology team can dissect the neck and perform the hypopharyngeal anastomosis most commonly via sewing or stapling [8, 10]. The conduit can be oriented isoperistaltic or anti-peristaltic; isoperistaltic is generally preferred based on greater peristaltic activity [20]. One or two-layers of interrupted suture have been utilized in other case reports; in our series, we used a two-layered approach [1–3, 15–17]. Less commonly, a stapler can be used which may be associated with faster operative times, fewer post-op wound healing complications, and shorter hospital stays [10].

Postoperatively, patients typically receive feeds from a J-tube placed during the procedure until there is confirmation of an intact anastomosis and the patient can tolerate a normal diet [1, 2, 14, 16, 17]. The anastomosis can be evaluated by barium swallow and upper endoscopy one to two weeks after surgery [5, 15]. Once the integrity of the anastomosis is verified, the patient’s diet can be slowly advanced [1]. Overall, patients typically stay in the hospital for two to three weeks after surgery [4, 9].

Follow-up is an important component for management of the multitude of complications associated with procedure. Complications after esophageal reconstruction with a hypopharyngeal anastomosis include leak, obstruction, stenosis, dehiscence, dysphagia, respiratory, infection, and death [1, 2, 4, 5, 9, 10, 12, 13, 15–17, 19, 21, 22]; a table depicting complications seen in the literature is shown in Table 3. In our case series, dysphagia occurred in all 3 patients. Case 1 developed dysphagia via stenosis of the hypopharyngeal anastomosis, which was treated with revision of the anastomosis. Other reports of revisional surgery are depicted in Table 4. Case 3 has required balloon dilation of the hypopharyngeal anastomosis x 6. As seen in the literature, dysphagia is not an abnormal outcome as patients can continue to form stenoses and scar tissue throughout their lives in cases of caustic ingestion [5, 15]. Compared to normal patients, colonic transit time is slower compared to normal adults given the less frequent colonic contractions and greater reliance on gravity [20]. Furthermore, Case 2’s dysphagia was due to delayed gastric emptying which was treated with endoscopic dilation of the pylorus and Botox injection. In addition to dysphagia, respiratory complications comprise a large proportion of complications in the literature; however, we did not witness any post-operative respiratory complications in our series [9, 12, 13, 15, 16]. Aspiration can occur via recurrent laryngeal nerve damage or poor swallowing; thus, it is important for patients to work with speech and swallow with gradual transition to a normal diet [2, 17]. Despite careful preservation of the laryngeal nerves in our series, Case 2 had temporary vocal cord paralysis that resolved a few weeks after surgery and Case 3 has dysphonia likely related to the extensive preoperative scar tissue formation from lye. Other potential complications include pneumothorax via tunneling of the conduit into the neck [5]; infection [12]; and cancer that could occur in the residual esophagus and stomach [14].

**Table 3:** Complications associated with a hypopharyngeal anastomosis, table design derived from Sanchez, et al. NR (Not Recorded); RFFF (Radial Forearm Free Flap).

Author	Period	Number of Patients with Hypopharyngeal / Pharyngeal Anastomosis	Anastomotic Leak	Obstruction	Stenosis or Stricture	Dehiscence	Dysphagia	Respiratory	Infection	Death	Other
Chilgar, et al	Jan 1994 to Dec 2012	94	3 (distal) 0 (proximal)	0	5	0	0	0	0	0	0
Jiang, et al	Aug 1988 to June 2023	14	4	0	1 prox, 1 distal	0	4	2	0	0	Disruption of the abdominal incision in 1
Wu, et al	Sept 1976 to April 2000	50	3 cervical, 2 abdominal	1 esophageal substitute, 3 intestinal	6 hypopharyngeal, 6 laryngotracheal	1 graft failure	8	1	0	1	Gastric mucocele with chronic anemia in 1
Lu, et al	March 2003 to May 2006	5	0	0	0	0	0	0	0	0	Tracheostomy stoma stenosis in 1
Tannuri and Tannuri	Feb 1982 to Dec 2015	19	0	0	9 (cervical)	1 (cologastric)	0	9	0	0	Diarrhea (10)
Denewer, et al	Jan 2004 to Dec 2012	142	NR	NR	Including 3 revisional surgeries: 26% pec; 4% jejunal; 0 augmented colon; 8.8% gastric pull up; 6.7% gastric tube	NR	NR	15	NR	15	Including 3 revisional surgeries: 4 cases of flap failure (3 jejunal, 1 pec) - Early fistula in 24% of pec, 8% jejunal, 25% augmented colon, 14.7% gastric pull up; 10% of gastric tube
Bisquera, et al	~ 2022	1 (oropharynx)	No complications	-	-	-	-	-	-	-	-
Pegan, et al	10-year period not specified	31	NR	NR	NR	3 with abdominal skin dehiscence in gastric tube recon group	3	NR	NR	0	Flap failure 3 and cutaneous fistula 1 in RFFF group
Tran and Celerier	1978–1986	18	NR	NR	9	NR	4	4	Necrosis in 1; phlebitis in 1	0 (3 died from malnutrition)	4 cervical and 1 abdominal fistula at digestive anastomosis
Marzouki, et al	~ 2021	1	No complications	-	-	-	-	-	-	-	-
Schultz, et al	~ 2018	8	-	-	1	-	3 pectoralis major	-	-	-	1 fistula in forearm group and 1 fistula in pec major
Wang, et al	May 1995 to Nov 2021	75 (mechanical in 28, manual in 47)	Mechanical in 2, manual in 13	NR	Mechanical in 2, Manual in 4	NR	NR	Mechanical in 5, manual in 15	Manual 8 vs Mechanical 0	Manual in 4	Post-op bleeding: 5 manual, 1 mechanical Tracheal fistula: 1 mechanical, 2 manual Cerebro-vascular complications 0 in mechanical, 2 in manual
Zeng, et al	Jan 2005 to March 2017	20	1	NR	0	NR	NR	NR	NR	0	NR
Choi, et al	~ 1997	7	NR	NR	3	NR	4	NR	NR	0	Redundant colon in 1, perforation in 1
Zangi, et al	2009 to 2014	9	-	-	-	-	4	7 (aspiration)	1 Sepsis from graft necrosis	3	2 vocal cord paralysis
Pesko, et al	Jan 1978 to Jan 2004	40	6	-	-	-	-	12	1 necrosis	6	Cardiovascular 2, Other 2
Inoue, et al	~ 1992	28	NR	-	-	-	-	-	-	-	-

**Table 4:** Indications for revisional surgery on the hypopharyngeal anastomosis during esophageal reconstruction, table design derived from Sanchez, et al. NR (Not recorded)

Author	Chilgar, et al	Jiang, et al	Wu, et al	Lu, et al	Tannuri and Tannuri	Denewer, et al	Bisquera, et al	Pegan, et al	Tran and Celerier	Marzouki, et al	Schultz, et al	Wang, et al	Zeng, et al	Choi, et al	Zangi, et al	Pesko, et al	Inoue, et al
**Period**	Jan 1994 to Dec 2012	Aug 1988 to June 2023	Sept 1976 to April 2000	March 2003 to May 2006	Feb 1982 to Dec 2015	Jan 2004 to Dec 2012	~ 2022	10-year period not specified	1978–1986	~ 2021	~ 2018	May 1995 to Nov 2021	Jan 2005 to March 2017	~ 1997	2009 to 2014	Jan 1978 to Jan 2004	~ 1992
**# Patients with hypopharyngeal anastomosis**	94	14	50	5	19	142	1 (oropharynx)	31	18	1	8	75	20	-	9	-	28
**Proximal Stricture**	NR	1	6 (5 had good result after revisional surgery)	No revisions	No revisions	-	No revisions	-	6 – unclear location of stenosis	No revisions	NR	6 (1 mechanical, 5 manual) required reoperation – unclear indication	NR	2	-	-	-
**Distal Stricture**	3 + 1	1	NR	0	-	-	-	-	-	**-**	**-**	**-**	**-**	**-**	**-**	**-**	**-**
**Redundant colon**	NR	1	NR	0	-	-	-	-	-	**-**	**-**	**-**	**-**	1	**-**	**-**	**-**
**Flap failure**	-	-	-	-	-	3	-	1	1 due to necrosis	**-**	**-**	**-**	**-**	**-**	**-**	**-**	**-**
**Perforation**	-	-	-	-	-	-	-	-	-	-	-	-	-	1	-	**-**	**-**
**Dysphagia**	-	-	-	-	-	-	-	-	-	-	-	-	-	-	2	-	**-**
**Leak**	-	-	-	-	-	-	-	-	-	-	-	-	-	-	-	2	**-**

The case series above describes successful esophageal reconstruction using a hypopharyngeal anastomosis using a two-layered interrupted suture technique to the left, lateral piriform sinus. All patients survived and have improved swallow function. 2 (66.7%) patients are tolerating a normal diet without supplemental feeds, which is similar or better than other studies in the literature [2, 4, 5, 9, 13, 15–17]. 2 (66.7%) patients developed dysphonia, which has been treated conservatively with speech and swallow. Additionally, our case series had no leaks, dehiscence, or respiratory complications.

## Supplementary Information

Below is the link to the electronic supplementary material.

Supplementary Material 1

## Data Availability

No datasets were generated or analysed during the current study.

## Abbreviations

J-tube: Jejunostomy Tube
NR: Not Recorded
PEG: Percutaneous Endoscopic Gastrostomy
RFFF: Radial Forearm Free Flap
RNY: Roux–en–Y
