# Supplementary material for: Esophageal reconstruction using a hypopharyngeal anastomosis – a single center experience with review of the literature
Source: J Cardiothorac Surg. 2025 Aug 26;20:345. doi: 10.1186/s13019-025-03575-8 (PMC12379545; doi:10.1186/s13019-025-03575-8)
Supplement: Supplementary file 1 — Supplementary Material 1 [file 13019_2025_3575_MOESM1_ESM.docx]

**Table 2.** General overview of hypopharyngeal anastomosis studies, table design derived from Sanchez, et al (2022). NR (Not recorded).

| **Author** | **Period** | **#**  **Patients with Hypopharyngeal Surgery** | **Method of Injury** | **Endoscopic Dilation** | **Time to Surgery from Injury** | **Conduit**  **(iso-peristaltic, anti-peristaltic)** | **Conduit perfusion** | **Route** | **Type of Anastomosis** | **Mortality** | **Tolerating normal diet?** | **Regain Swallow**  **Function?** | **Still using feeding tube?** |
| --- | --- | --- | --- | --- | --- | --- | --- | --- | --- | --- | --- | --- | --- |
| Chilgar, et al | Jan 1994 to December 2012 | 94 | Hypopharyngeal Cancer, Squamous cancer | NR | NR | Free colon or ileo-colon iso | NR | NR | 2-layered suture vs. modified 2-layer suture with distal conical trimming | NR | NR | NR | NR |
| Jiang, et al | Aug 1988 to June 2023 | 14 | Caustic injuries | NR | 17.5 months | Left colon, anti | Mesenteric angiography, temporary occlusion | Mediastinum | 1-layered suture technique (prox)  2-layered suture technique (distal) | NR | 14/14 | NR | NR |
| Wu, et al | Sept 1976 to April 2000 | 50 | Caustic injuries | NR | 37 (74%) operated on within 6 months | 28/50 with ileocolon | Temporary clamping | 44/50 substernal | 1 or 2-layered interrupted sutures above level of abnormal mucosa | 1/50 | NR | 42/50 | NR |
| Lu, et al | March 2003 to May 2006 | 5 | Stage IVA hypopharynx cancer | NR | NR | Left colon with distal arterial enhancement (superior thyroid artery in 4; facial artery in 1) | Visual | Posterior mediastinum | NR | 0 | All | All | NR |
| Tannuri and Tannuri | Feb 1982 to Dec 2015 | 19 | Caustic | Yes | NR | Transverse and right colon; isoperistaltic | NR | Retrosternal, unless esophagectomy done where posterior mediastinum was used | 1-layer interrupted | 0 | All | All | All |
| Denewer, et al | Jan 2004 to Dec 2012 | 142 | Most squamous cell | NR | NR | Including 3 revisional surgeries; pectoralis flap in 48, jejunal flap in 28, augmented colon bypass in 4, gastric pull-up in 32, gastric tube in 30 | NR | NR | NR | 15/142 | Including 3 revisional surgeries: 40% of the pectoralis flap; 80% jejunal flap; 75% of augmented colon bypass; 82.4% with gastric pull-up; 86.7% with gastric tube | NR | NR |
| Bisquera, et al | ~2022 | 1 (oropharynx) | Caustic ingestion | No- complete obliteration | ~6 months | Left colon | Temporary clamping | Subcutaneous | Two-layered suture technique proximal; Roux-en-Y | No | Yes | Yes | No |
| Pegan, et al | 10-year period | 31 | Hypopharyngeal carcinoma | 0 | NR | Radial forearm flap in 9; jejunum in 7, gastric tube in 15 | NR | NR | NR | 0% | 29/31 | 29/31 | 2/31 |
| Tran and Celerier | 1978-1986 | 18 | Caustic | NR | 4 months- 28 years | Right ileocolon in 17; transverse colon in 1 | NR | Retrosternal | NR | 0 (3 died from severe malnutrition) | 11 | 11 | 4/14 |
| Marzouki, et al | ~2021 | 1 | Hypopharyngeal cancer, squamous cell | NR | NR | Tubed radial forearm flap | NR | NR | NR | No | Yes | Yes | No |
| Schultz, et al | ~2018 | 8 | NR | NR | NR | Forearm flap and tubed pectoralis major flap | NR | NR | Prox: Vicryl suture; Distal: Vicryl suture and 21mm diameter circular stapler with 2 rows of staples | 0 | NR | 5/5 forearm | NR |
| Wang, et al | May 1995 to Nov 2021 | 75 | Hypopharyngeal cancer | NR | NR | Stomach | NR | Posterior Mediastinum | Manual in 47; mechanical in 28 via circular stapler | 4 | NR | NR | NR |
| Zeng, et al | Jan 2005 to March 2017 | 20 | Corrosive | NR | 6 months to 45 years | Colon | Palpation temporary occlusion | Retrosternal | End-to-end manner with single-layer and broad-border hand sewn with delayed absorbable suture; distal end with continuous suture | 0 | NR | NR | NR |
| Choi, et al | ~1997 | 7 | Alkali and acid | Yes | 6 months-10 years | Colon, radial forearm flap, right shoulder pedicle flap | NR | NR | NR | 0 | 3 | 5 | 4 |
| Zangi, et al | 2009 to 2014 | 9 | Corrosive | NR | 4-10 months | Left colon isoperistaltic | Temporary clamping | Retrosternal or Posterior Mediastinum | Single layer of interrupted Vicryl | 3 | NR | 6 | NR |
| Pesko, et al | January 1978 to January 2004 | 40 | Hypopharyngeal carcinoma | NR | NR | Stomach in 29; colon in 11 | NR | NR | NR | 6 (13%) | NR | NR | NR |
| Inoue, et al | ~1992 | 28 | Hypopharyngeal carcinoma | NR | NR | Jejunum with microvascular anastomosis | Angiography preop | NR | NR | NR | NR | NR | NR |

**Table 3.** Complications associated with a hypopharyngeal anastomosis, table design derived from Sanchez, et al. NR (Not Recorded); RFFF (Radial Forearm Free Flap).

| **Author** | **Period** | **Number of Patients with Hypopharyngeal / Pharyngeal Anastomosis** | **Anastomotic Leak** | **Obstruction** | **Stenosis or Stricture** | **Dehiscence** | **Dysphagia** | **Respiratory** | **Infection** | **Death** | **Other** |
| --- | --- | --- | --- | --- | --- | --- | --- | --- | --- | --- | --- |
| Chilgar, et al | Jan 1994 to Dec 2012 | 94 | 3 (distal)  0 (proximal) | 0 | 5 | 0 | 0 | 0 | 0 | 0 | 0 |
| Jiang, et al | Aug 1988 to June 2023 | 14 | 4 | 0 | 1 prox, 1 distal | 0 | 4 | 2 | 0 | 0 | Disruption of the abdominal incision in 1 |
| Wu, et al | Sept 1976 to April 2000 | 50 | 3 cervical, 2 abdominal | 1 esophageal substitute, 3 intestinal | 6 hypopharyngeal, 6 laryngotracheal | 1 graft failure | 8 | 1 | 0 | 1 | Gastric mucocele with chronic anemia in 1 |
| Lu, et al | March 2003 to May 2006 | 5 | 0 | 0 | 0 | 0 | 0 | 0 | 0 | 0 | Tracheostomy stoma stenosis in 1 |
| Tannuri and Tannuri | Feb 1982 to Dec 2015 | 19 | 0 | 0 | 9 (cervical) | 1 (cologastric) | 0 | 9 | 0 | 0 | Diarrhea (10) |
| Denewer, et al | Jan 2004 to Dec 2012 | 142 | NR | NR | Including 3 revisional surgeries: 26% pec; 4% jejunal; 0 augmented colon; 8.8% gastric pull up; 6.7% gastric tube | NR | NR | 15 | NR | 15 | Including 3 revisional surgeries: 4 cases of flap failure (3 jejunal, 1 pec)  - Early fistula in 24% of pec, 8% jejunal, 25% augmented colon, 14.7% gastric pull up; 10% of gastric tube |
| Bisquera, et al | ~2022 | 1 (oropharynx) | No complications | - | - | - | - | - | - | - | - |
| Pegan, et al | 10-year period not specified | 31 | NR | NR | NR | 3 with abdominal skin dehiscence in gastric tube recon group | 3 | NR | NR | 0 | Flap failure 3 and cutaneous fistula 1 in RFFF group |
| Tran and Celerier | 1978-1986 | 18 | NR | NR | 9 | NR | 4 | 4 | Necrosis in 1; phlebitis in 1 | 0 (3 died from malnutrition) | 4 cervical and 1 abdominal fistula at digestive anastomosis |
| Marzouki, et al | ~2021 | 1 | No complications | - | - | - | - | - | - | - | - |
| Schultz, et al | ~2018 | 8 | - | - | 1 | - | 3 pectoralis major | - | - | - | 1 fistula in forearm group and 1 fistula in pec major |
| Wang, et al | May 1995 to Nov 2021 | 75 (mechanical in 28, manual in 47) | Mechanical in 2, manual in 13 | NR | Mechanical in 2, Manual in 4 | NR | NR | Mechanical in 5, manual in 15 | Manual 8 vs Mechanical 0 | Manual in 4 | Post-op bleeding: 5 manual, 1 mechanical  Tracheal fistula: 1 mechanical, 2 manual  Cerebro-vascular complications 0 in mechanical, 2 in manual |
| Zeng, et al | Jan 2005 to March 2017 | 20 | 1 | NR | 0 | NR | NR | NR | NR | 0 | NR |
| Choi, et al | ~1997 | 7 | NR | NR | 3 | NR | 4 | NR | NR | 0 | Redundant colon in 1, perforation in 1 |
| Zangi, et al | 2009 to 2014 | 9 | - | - | - | - | 4 | 7 (aspiration) | 1 Sepsis from graft necrosis | 3 | 2 vocal cord paralysis |
| Pesko, et al | Jan 1978 to Jan 2004 | 40 | 6 | - | - | - | - | 12 | 1 necrosis | 6 | Cardiovascular 2, Other 2 |
| Inoue, et al | ~1992 | 28 | NR | - | - | - | - | - | - | - | - |

**Table 4.** Indications for revisional surgery on the hypopharyngeal anastomosis during esophageal reconstruction, table design derived from Sanchez, et al. NR (Not recorded).

| **Author** | **Chilgar, et al** | **Jiang, et al** | **Wu, et al** | **Lu, et al** | **Tannuri and Tannuri** | **Denewer, et al** | **Bisquera, et al** | **Pegan, et al** | **Tran and Celerier** | **Marzouki, et al** | **Schultz, et al** | **Wang, et al** | **Zeng, et al** | **Choi, et al** | **Zangi, et al** | **Pesko, et al** | **Inoue, et al** |
| --- | --- | --- | --- | --- | --- | --- | --- | --- | --- | --- | --- | --- | --- | --- | --- | --- | --- |
| **Period** | Jan 1994 to Dec 2012 | Aug 1988 to June 2023 | Sept 1976 to April 2000 | March 2003 to May 2006 | Feb 1982 to Dec 2015 | Jan 2004 to Dec 2012 | ~2022 | 10-year period not specified | 1978-1986 | ~2021 | ~2018 | May 1995 to Nov 2021 | Jan 2005 to March 2017 | ~1997 | 2009 to 2014 | Jan 1978 to Jan 2004 | ~1992 |
| **# Patients with hypopharyngeal anastomosis** | 94 | 14 | 50 | 5 | 19 | 142 | 1 (oropharynx) | 31 | 18 | 1 | 8 | 75 | 20 | - | 9 | - | 28 |
| **Proximal Stricture** | NR | 1 | 6 (5 had good result after revisional surgery) | No revisions | No revisions | - | No revisions | - | 6 – unclear location of stenosis | No revisions | NR | 6 (1 mechanical, 5 manual) required reoperation – unclear indication | NR | 2 | - | - | - |
| **Distal Stricture** | 3+1 | 1 | NR | 0 | - | - | - | - | - | **-** | **-** | **-** | **-** | **-** | **-** | **-** | **-** |
| **Redundant colon** | NR | 1 | NR | 0 | - | - | - | - | - | **-** | **-** | **-** | **-** | 1 | **-** | **-** | **-** |
| **Flap failure** | - | - | - | - | - | 3 | - | 1 | 1 due to necrosis | **-** | **-** | **-** | **-** | **-** | **-** | **-** | **-** |
| **Perforation** | - | - | - | - | - | - | - | - | - | - | - | - | - | 1 | - | **-** | **-** |
| **Dysphagia** | - | - | - | - | - | - | - | - | - | - | - | - | - | - | 2 | - | **-** |
| **Leak** | - | - | - | - | - | - | - | - | - | - | - | - | - | - | - | 2 | **-** |
